# Supplementary material for: Causal Relationship between Aging and Anorexia Nervosa: A White-Matter-Microstructure-Mediated Mendelian Randomization Analysis
Source: Biomedicines. 2024 Aug 16;12(8):1874. doi: 10.3390/biomedicines12081874 (PMC11351342; doi:10.3390/biomedicines12081874)
Supplement: Supplementary file 1 [file biomedicines-12-01874-s001.zip › Supplementary Figure/Figure S4/Singe SNP plot of MR analysis for TL to ubm-b-1619.pdf]

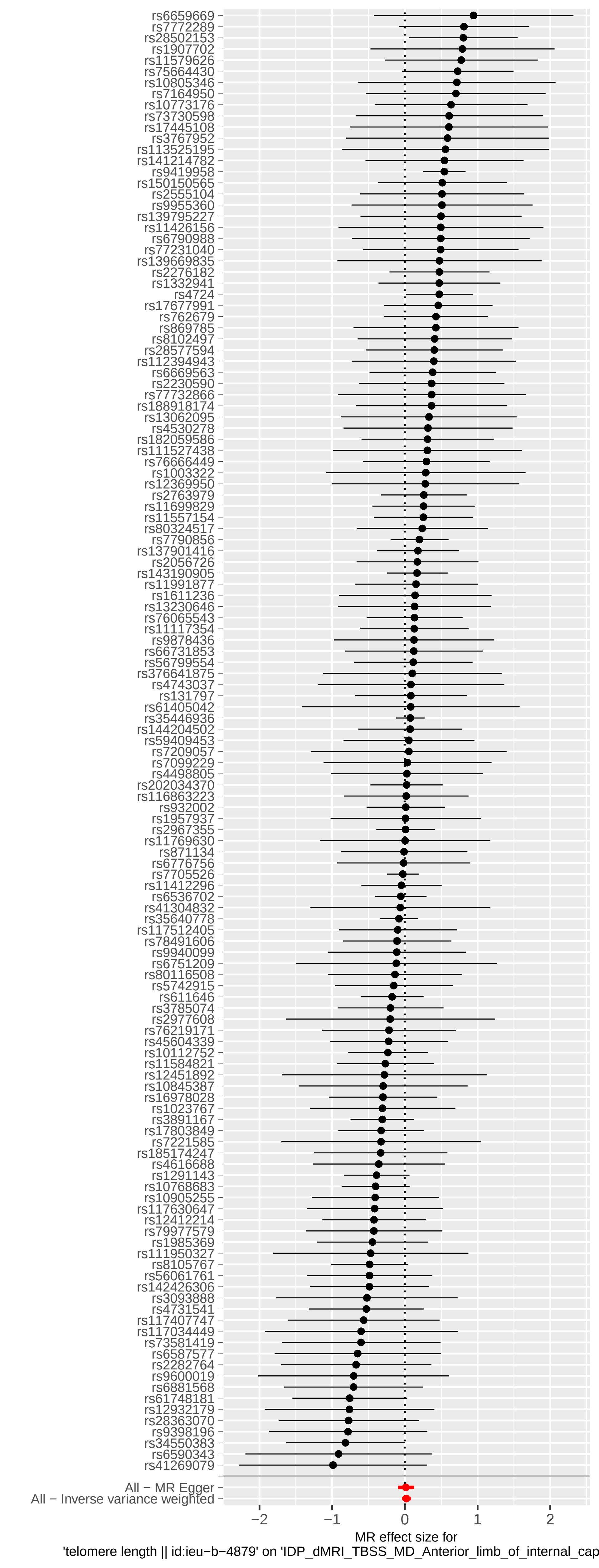
rs6659669
rs7772289
rs28502153
rs1907702
rs11579626
rs75664430
rs10805346
rs7164950
rs10773176
rs73730598
rs17445108
rs3767952
rs113525195
rs141214782
rs9419958
rs150150565
rs2555104
rs9955360
rs139795227
rs11426156
rs6790988
rs77231040
rs139669835
rs2276182
rs1332941
rs4724
rs17677991
rs762679
rs869785
rs8102497
rs28577594
rs112394943
rs6669563
rs2230590
rs77732866
rs188918174
rs13062095
rs4530278
rs182059586
rs111527438
rs76666449
rs1003322
rs12369950
rs2763979
rs11699829
rs11557154
rs80324517
rs7790856
rs137901416
rs2056726
rs143190905
rs11991877
rs1611236
rs13230646
rs76065543
rs11117354
rs9878436
rs66731853
rs56799554
rs376641875
rs4743037
rs131797
rs61405042
rs35446936
rs144204502
rs59409453
rs7209057
rs7099229
rs4498805
rs202034370
rs116863223
rs932002
rs1957937
rs2967355
rs11769630
rs871134
rs6776756
rs7705526
rs11412296
rs6536702
rs41304832
rs35640778
rs117512405
rs78491606
rs9940099
rs6751209
rs80116508
rs5742915
rs611646
rs3785074
rs2977608
rs76219171
rs45604339
rs10112752
rs11584821
rs12451892
rs10845387
rs16978028
rs1023767
rs3891167
rs17803849
rs7221585
rs185174247
rs4616688
rs1291143
rs10768683
rs10905255
rs117630647
rs12412214
rs79977579
rs1985369
rs111950327
rs8105767
rs56061761
rs142426306
rs3093888
rs4731541
rs117407747
rs117034449
rs73581419
rs6587577
rs2282764
rs9600019
rs6881568
rs61748181
rs12932179
rs28363070
rs9398196
rs34550383
rs6590343
rs41269079
All − MR Egger
All − Inverse variance weighted
−2
−1
0
1
2
MR effect size for
'telomere length || id:ieu−b−4879' on 'IDP_dMRI_TBSS_MD_Anterior_limb_of_internal_cap
